# The role of targeted therapy and immune therapy in the management of non-small cell lung cancer brain metastases

**DOI:** 10.3389/fonc.2023.1110440

**Published:** 2023-02-23

**Authors:** Cole Billena, Mina Lobbous, Christine A. Cordova, David Peereboom, Alejandro Torres-Trejo, Timothy Chan, Erin Murphy, Samuel T. Chao, John Suh, Jennifer S. Yu

**Affiliations:** ^1^ Department of Radiation Oncology, Cleveland Clinic Foundation, Cleveland, OH, United States; ^2^ Brain Tumor and Neuro-Oncology Center, Cleveland Clinic Foundation, Cleveland, OH, United States; ^3^ Center for Cancer Stem Cell Biology, Department of Cancer Biology, Cleveland Clinic Foundation, Cleveland, OH, United States

**Keywords:** brain metastases, targeted therapy, immunotherapy, non-small cell lung (NSCLC), EGFR, ALK, radiation, stereotactic radiosurgery (SRS)

## Abstract

Brain metastases are a significant source of morbidity and mortality in patients with non-small cell lung cancer. Historically, surgery and radiation therapy have been essential to maintaining disease control within the central nervous system due to poorly penetrant conventional chemotherapy. With the advent of targeted therapy against actionable driver mutations, there is potential to control limited and asymptomatic intracranial disease and delay local therapy until progression. In this review paper, intracranial response rates and clinical outcomes to biological and immune therapies are summarized from the literature and appraised to assist clinical decision making and identify areas for further research. Future clinical trials ought to prioritize patient-centered quality of life and neurocognitive measures as major outcomes and specifically stratify patients based on mutational marker status, disease burden, and symptom acuity.

## Introduction

Lung cancer is the leading cause of cancer-related deaths and the second most common cancer in men and women in the United States (1). Unfortunately, brain metastases (BM) remain a salient problem with nearly 20% of all BM occurring in patients with lung cancers (2). The vast majority of lung cancer cases comprise of non-small cell lung cancers (NSCLC) (3). Advances in systemic therapy have improved median survival of the most favorable metastatic NSCLC with BM to more than four years (4). Recent updates to the validated diagnosis-specific graded prognostic assessment (DS-GPA) for lung adenocarcinoma have included EGFR, ALK, and PD-L1 status to the scoring criteria (5, 6). These impressive results have brought about a rethinking of BM management and the integration of systemic therapy with current local therapy options.

Classically, local therapy including surgical resection, whole brain radiation therapy (WBRT), and stereotactic radiosurgery (SRS) has been integral to controlling CNS disease due to the morbidity of BM, poor CNS penetration by conventional chemotherapy, and the nearly ubiquitous exclusion of metastatic CNS involvement in clinical trials (7, 8). Seminal papers by Patchell et al. demonstrated an overall survival and quality of life benefit to surgical resection of a single BM(9). and reduction of CNS recurrence and neurologic death with adjuvant WBRT (10). However, WBRT has been associated with impaired cognition and quality of life. Memantine and hippocampal avoidance have emerged as strategies to mitigate these toxicities (11–13). Additionally, SRS has gained favor as definitive and adjuvant therapy in lieu of surgical resection and WBRT due to reduced neurocognitive sequelae (14–17).

Although CNS penetration by newer systemic therapy options has been reported and clinical efficacy is an area of active study, their integration into the current paradigm of BM management continues to evolve for different clinical scenarios. Guidelines jointly published by ASCO-SNO-ASTRO presently recommend local therapy for symptomatic brain metastases (18). However, after multidisciplinary review, deferral may be offered to patients with asymptomatic brain metastases with either EGFR-mutant NSCLC receiving osimertinib or icotinib, or ALK rearranged NSCLC receiving alectinib, brigatinib, or ceritinib (18).

In this review, we aim to describe the intracranial activity of systemic therapies, outcomes of clinical trials using combinations of systemic and local therapy, and identify areas for future study to guide clinical practice. It is possible that radiation therapy (RT) and surgery may be omitted if systemic therapy sufficiently controls limited and asymptomatic intracranial disease. Radiation and systemic therapy may prove synergistic to enhance control of targeted lesions. The need for WBRT may be obviated if systemic therapy eliminates microscopic intracranial disease, allowing for combinatorial SRS and systemic therapy options.

## Epidermal growth factor receptor inhibitors

Epidermal growth factor receptor (EGFR) is a receptor tyrosine kinase whose activation increases cell survival and proliferation (19). EGFR is mutated in 10-15% of all NSCLC cases, and 90% of all EGFR mutations comprise of exon 19 deletion and exon 21 L858R point mutations (20). Epidemiologically, EGFR mutations are more prevalent in Asian compared to Caucasian populations and are associated with non-smoking patient history (21). This is in contrast to smoking-associated NSCLC in which EGFR mutations are rare. Interestingly, the risk of BM seems to be 2-3 times higher in EGFR-mutated lung cancers compared to wildtype, and one third of these patients will develop intracranial progression sometime during their disease course, underscoring high neurotropism of these EGFR-mutant tumors (22–24). 

EGFR tyrosine kinase inhibitors (EGFR TKIs) were initially explored in the chemo-refractory setting (25). but it was quickly realized that the presence of mutations was predictive of clinical response (26). First generation EGFR TKIs, which reversibly bind to the tyrosine kinase domain of EGFR, include gefinitib, erlotinib, and icotinib. Analysis of plasma and cerebrospinal fluid (CSF) concentrations of gefinitib and erlotinib show sufficient CNS penetration to elicit clinical responses (27). Overall, intracranial objective response rates (icORR) of BM in patients with EGFR-mutated NSCLC for erlotinib and gefitinib largely range from 60%-80%, and disease control rates are higher at 80-100% (**Table 1**) (28–35). Median progression-free survival (PFS) ranged from 6 months to 15 months. The majority of data is drawn from retrospective series, with one of the larger series by Zhang et al. of 43 patients with EGFR-mutated NSCLC with BM treated with erlotinib or gefitinib demonstrating an icORR of 57% and intracranial disease control rate (icDCR) of 91% (38). In the phase III FLAURA trial, use of erlotinib and gefinitib within the control arm showed similar results with an icORR of 68% and icDCR of 89% (43). Investigations into pulse-dose erlotinib to overcome TKI resistance and increase CNS penetration report icORR of 67%-74% and icDCR of 78-100%, but in the setting of patients with TKI pretreatment, median time to CNS progression was short at 2.7 months (30–32). Icotinib is another first- generation TKI that is approved in China for advanced EGFR-mutated NSCLC. Icotinib was found to have similar intracranial efficacy to gefitinib (37). In a phase III trial (BRAIN) comparing icotinib versus WBRT, icotinib had an icORR of 65%, icDCR of 85%, and a median intracranial PFS of 10 months, outcomes superior to WBRT alone (44).

**Table 1:** CNS-related Clinical Outcomes in EGFR-mutated NSCLC with Brain Metastases.

Study	Population	N	Intervention	icORR	icDCR	mPFS (mo)	mTTP (mo)	mDOR (mo)
(28)	EGFR mutated NSCLC with BM	9	Erlotinib	NA	80%	NA	NA	NA
(29)	EGFR mutated NSCLC with BM	17	Erlotinib	82.40%	NA	NA	11.7	NA
(30)	EGFR mutated NSCLC with BM with prior TKI	9	Pulse dose erlotinib	67%	78%	NA	2.7	NA
(31)	EGFR mutated NSCLC with BM, TKI-naive	11	Pulse dose erlotinib	NA	100%	NR	NA	NA
(32)	EGFR mutated NSCLC with BM, TKI-naive	19	Pulse dose erlotinib	74%	84%	9.7	NA	NA
(33)	EGFR mutated NSCLC with BM	4	erlotinib	50%	NA	NA	NA	NA
(34)	EGFR mutated NSCLC with BM previously on gefitinib	6	erlotinib	50%	100%	NA	NA	NA
(35)	EGFR mutated NSCLC with BM	15	Gefitinib	60%	NA	8.7	NA	NA
(36)	EGFR mutated NSCLC with BM	41	Gefinitib	87.80%	NA	14.5	NA	NA
(37)	EGFR mutated NSCLC with BM	22	Gefinitib	59%	81.8	10.6	NA	NA
(21, 38)	EGFR mutated NSCLC with BM	43	erlotinib or gefitinib	57%	91%	9.3	NA	NA
(39)	EGFR mutated NSCLC with BM	28	erlotinib or gefitinib	83%	93%	6.6	NA	NA
FLAURA	EGFR mutated NSCLC with BM, treatment-naïve	19	erlotinib or gefitinib	68%	89%	NA	NA	NA
(37)	EGFR mutated NSCLC with BM	22	Icotinib	67%	59.1	8.4	NA	NA
BRAIN	EGFR mutated NSCLC with BM, treatment-naïve	85	Icotinib	65%	85%	10	NA	NA
(40)	EGFR mutated NSCLC with BM, s/p first gen TKI	31	afatinib	35%	66%	NA	NA	NA
(41)	EGFR mutated NSCLC with BM, treatment-naïve	74	afatinib	81.10%	95.90%	NA	NA	NA
(42)	EGFR mutated NSCLC with BM, treatment-naïve	28	afatinib	85.70%	89.30%	NA	NA	NA
FLAURA	EGFR mutated NSCLC with BM, treatment-naïve	41	osimertinib	91%	95%	NR	NA	NA
Pooled AURA/AURA2	EGFR mutated NSCLC with BM, T790M-positive and previously treated with EGFR-TKI	50	osimertinib	54%	92%	NR	NA	NA
AURA3	EGFR mutated NSCLC with BM, T790M-positive and previously treated with EGFR-TKI	46	osimertinib	70%	NA	11.7	NA	NA
OCEAN	EGFR mutated NSCLC with BM, treatment-naïve	39	osimertinib	70%	NA	7.1	NA	NA

NSCLC, non-small cell lung cancer; BM, brain metastases; icORR, intracranial overall response rate; icDCR, intracranial disease control rate; mo, month; TKI, tyrosine kinase inhibitor; mTTP, median time to progression; mPFS, median progression free survival; mDOR, median duration of response; NR, not reached; RT, radiation therapy; NA, not applicable.

Afatinib is a second generation, irreversible EGFR-TKI inhibitor that binds to multiple family members including EGFR, HER2,and HER4 (45). A prospective trial conducted by Tamiya et al. confirmed its CNS penetration (46). In the treatment-naïve setting, response rates from retrospective data appear excellent. Wei et al. reported icORR and icDCR of 81.1% and 95.9%, respectively, in a cohort of 74 patients with EGFR-mutated NSCLC, while Li et al. similarly note icORR and icDCR of 85.7% and 89.3%, respectively, in 28 patients (41, 42). In the setting of prior TKI treatment, however, response rates appear significantly lower as shown by Hoffknecht et al. with icORR of 35% and icDCR of 66% in 31 patients previously treated with a first generation TKI (40).

Osimertinib is a third generation, irreversible EGFR-TKI approved for the first-line treatment of EGFR-mutant metastatic NSCLC. A subgroup analysis of the FLAURA trial (FLAURA), which demonstrated superiority of osimertinib over first generation EGFR-TKIs in advanced EGFR-mutated NSCLC, examined outcomes of 128 patients with CNS lesions. Among the 41 patients with measurable CNS lesions, excellent icORR and icDCR of 91% and 95% respectively were found, which notably surpassed outcomes for gefitinib and erlotinib as mentioned above (43). In a pooled analysis of the phase I AURA and phase II AURA2 trials that examined osimertinib in patients with the EGFR T790M acquired resistance mutation following prior treatment with EGFR-TKIs, icORR was 54%, not as high in the treatment naïve setting, but icDCR was impressive at 92% (47). Median PFS was not reached with a follow-up time of 11 months. Consistent with the high rates of CNS response rates, molecular imaging with 11C-labelled osimertinib in a phase I study (ODIN-BM) showed excellent blood-brain-barrier penetration (48).

Due to the high CNS efficacy of several EGFR-TKI, particularly the newer generation TKIs, delaying immediate radiation for BM may be warranted for select patients with asymptomatic or low burden of BM. In a retrospective series of 17 patients with EGFR mutant NSCLC, Porta et al. found that among the 8 patients who received erlotinib without WBRT had a satisfactory icORR of 75% compared to the icORR of 88% for the patients receiving erlotinib plus WBRT (29). In a cohort of 41 patients receiving gefinitib without prior RT, Iuchi et al. noted 48.8% ultimately received salvage RT with a median time to RT of 17.9 months (36). Additionally, Park et al. found that erlotinib or gefinitib provided a local therapy-free survival of 12.6 months in a prospective phase II study of 28 patients (39). A multi-institutional retrospective study by Thomas et al. evaluating both EGFR and ALK TKIs with or without RT for BM included 95 patients with EGFR+ NSCLC and found no difference in time to intracranial progression to time to treatment failure (49). These data suggest that for select patients with BM, treatment with brain-penetrant EGFR-TKI may be considered.

The BRAIN trial was a phase III randomized study comparing icotinib versus WBRT for patients with at least 3 brain lesions and naïve to EGFR-TKIs and RT (44). This trial demonstrated the superiority of icotinib over WBRT, with median intracranial PFS of 10 months versus 4.8 months, respectively, although it did not translate into improvements in overall survival or neurologic symptomology. Interestingly, a retrospective multi-institutional prospective cohort study of 351 patients found that delaying radiation decreased overall survival rates (50). Upfront EGFR-TKI followed by SRS at the time of intracranial progression had a median overall survival of 25 months compared to 46 months with upfront SRS followed by EGFR-TKI and 30 months with upfront WBRT followed by EGFR-TKI. Additionally, median intracranial PFS was notably higher in the upfront RT arms (37.9 months) versus upfront EGFR-TKI arm (10.6 months), and 58% of patients receiving upfront EGFR-TKIs ultimately required salvage RT for CNS progression. Notably, the vast majority of the cohort (98%) received erlotinib. Given the improved CNS efficacy of third generation osimertinib, the results of the phase II OUTRUN (NCT03497767) comparing osimertinib versus osimertinib with SRS in patients with EGFR-mutated NSCLC with the primary endpoint of intracranial PFS are eagerly awaited.

## Anaplastic lymphoma kinase inhibitors

Anaplastic lymphoma kinase (ALK) is a receptor tyrosine kinase, initially discovered in anaplastic large cell lymphoma, but chromosomal rearrangements resulting in a fusion gene, most commonly with echinoderm microtubule-associated protein-like 4 (EML4), occur in 3-7% of NSCLC (51, 52). ALK rearrangements are associated with the adenocarcinoma subtype, female sex, young age, no or light smoking history, and are mutually exclusive with EGFR and KRAS mutations (53). Patients with ALK-positive NSCLC have a higher risk of BM, with 20-30% of patients having BM at diagnosis (54, 55).

Crizotinib is a first-generation multi-target TKI targeting ALK as well as c-MET and ROS1. Overall CNS activity appears to be modest in *post hoc* analysis of clinical trials (**Table 2**). The CNS has been recognized as a sanctuary site with ALK TKI penetration rates as low as 0.26% (66, 67). In the pooled analysis of PROFILE 1005 and 1007, Costa et al. found that among the 275 patients with ALK+ NSCLC and BM who progressed after at least one line of systemic therapy, icORR, icDCR, and median time to progression of intracranial disease were 33%, 62%, and 12.3 months, respectively, for those previously treated with radiation, and 18%, 62% and 7 months, respectively, for those untreated (68). The authors noted 70% of progression events in patients with existing BM occur in the CNS and approximately 20% of patients without BM eventually developed CNS disease. Comparatively, in the upfront setting, a review of 39 treatment naïve patients with ALK+ NSCLC with BM in the PROFILE 1014 study showed improved results with an icORR of 77%, icDCR of 85% and 56% at 12 weeks and 24 weeks, respectively, and time to intracranial progression of 15.7 months (69). In several phase III trials evaluating next generation ALK-TKIs, crizotinib was used as the control arm and demonstrated relatively poor icORRs ranging from 21% to 50% and duration of responses lasting 5 to 10 months (70–73)

**Table 2:** CNS-related Clinical Outcomes in ALK-positive NSCLC with Brain Metastases.

Study	Population	N	Intervention	icORR	icDCR	mPFS (mo)	mTTP (mo)	mDOR (mo)
Pooled PROFILE 1005/1007 (Costa et al.)	ALK positive NSCLC with BM, s/p at least 1 line of systemic therapy, prior RT or untreated (no prior RT)	275	crizotinib	Untreated: 18%, Treated: 33%	Untreated: 56%, RT-rreated: 62%	NA	NA	Untreated: mDOR 7 months, RT-treated: mDOR 12.3 months
PROFILE 1014	ALK positive NSCLC with BM, treatment naive	39	crizotinib	77%	56%	9	15.7	NA
ALEX	ALK positive NSCLC with BM, treatment naive	22	crizotinib	50%	NA	NA	NA	5.5
ALTA-1L	ALK positive NSCLC with BM, ALKi naïve	21	crizotinib	29%	NA	NA 9.2	NA	9.2
eXalt3	ALK positive NSCLC with BM, ALKi naïve	19	crizotinib	21.10%	NA	7.5	NA	NA
CROWN	ALK positive NSCLC with BM, treatment naive	13	crizotinib	23%	NA	NA	NA	10.2
eXalt3	ALK positive NSCLC with BM, ALKi naïve	11	ensartinib	64%	100%	11.8	NA	NA
(56)	ALK positive NSCLC with BM, with or without prior ALKi	14	ensartinib	64.30%	92.90%	NA	NA	5.7
(57)	ALK positive NSCLC with BM, s/p crizotinib	40	ensartinib	70%	98%	NA	NA	NA
(58)	ALK positive NSCLC with BM, s/p crizotinib	44	brigatinib	90 mg dose: 42%, 180 mg dose 67%	NA	90 mg dose: 15.6, 180 mg dose: 12.8	NA	NA
ALTA-1L	ALK positive NSCLC with BM, ALKi naïve	18	brigatinib	78%	NA	NA	NA	27.9
ASCEND 1	ALK positive NSCLC with BM, heavily pretreated with ALKi and treatment naïve	94	ceritinib	Untreated: 72.3%, Pretreated: 56.4%	Untreated: 78.9%, Treated 65.3%	Untreated: 18.4, Pretreated: 6.9	NA	NA
ASCEND 2	ALK positive NSCLC with BM, s/p platinum therapy and crizotinib	20	ceritinib	45%	80%	NA	NA	NA
ASCEND 4	ALK positive NSCLC with BM, untreated	22	ceritinib	72.70%	86.30%		NA	16.6
ASCEND 5	ALK positive NSCLC with BM, s/p platinum agent and crizotinib	17	ceritinib	35%	NA	NA	NA	6.9
ASCEND 7	ALK positive NSCLC with BM, +/- prior ALKi +/- prior RT	138	ceritinib	prior RT + ALKi: 39.3%, prior ALKi only: 27.6%, prior RT only: 28.6%, RT/ALKi naïve: 51.5%	NA	NA	NA	NA
ALEX	ALK positive NSCLC with BM, treatment naive	21	alectinib	81%	NA	NA	NA	17.3
(59)	ALK positive NSCLC with BM, s/p crizotinib	34	alectinib	58.80%	85.30%	NA	NA	11.1
(60)	ALK positive NSCLC with BM, s/p crizotinib	16	alectinib	75%	100%	NA	NA	11.1
(61)	ALK positive NSCLC with BM, s/p crizotinib	21	alectinib	52%	90%	NA	NA	NA
ALUR	ALK positive NSCLC with BM, s/p platinum agent and crizotinib	24	alectinib	54.20%	79.20%	NR	NA	NA
(62)	ALK positive NSCLC with BM, s/p crizotinib	35	alectinib	57%	83%	NA	NA	10.3
CROWN	ALK positive NSCLC with BM, treatment naive	17	Lorlatinib	82%	NA	NA	NA	NA
(63)	ALK positive NSCLC with BM, s/p prior ALK TKI	81	lorlatinib	S/p crizotinib: 87.5%, S/p 1 or more 2nd Gen TKI: 54.4%	S/p crizotinib: 100%, S/p 1 or more 2nd Gen TKI: 84.2%	NA	NA	S/p crizotinib: NR, S/p 1 or more 2nd Gen TKI: 12.4
(64)	ALK positive NSCLC with BM, s/p prior 2nd gen TKI	57	lorlatinib	S/p 1 prior 2nd gen TKI: 66.7%, s/p 2 or more 2nd gen TKI: 54.2%	S/p 1 prior 2nd gen TKI: 66.6%, s/p 2 or more 2nd gen TKI: 87.4%	NA	NA	S/p 1 prior 2nd gen TKI: 20.7, s/p 2 or more 2nd gen TKI: 12.4
(65)	ALK positive NSCLC with BM, s/p 2nd gen ALK TKI with CNS-only progression	23	lorlatinib	59%	95%	24.6	NA	NA

NSCLC, non-small cell lung cancer; BM, brain metastases; icORR, intracranial overall response rate; icDCR, intracranial disease control rate; mo, month; TKI, tyrosine kinase inhibitor; mTTP, median time to progression; mPFS, median progression free survival; mDOR, median duration of response; NR, not reached; RT, radiation therapy; NA, not applicable.

The eventual emergence of resistance to crizotinib *via* secondary mutations and alternative signaling pathways led to the development of second generation ALK TKIs (74). Ceritinib is a highly potent ALK TKI active against several crizotinib-resistance mutations including the gatekeeper L1196M mutation. Ceritinib is more selective than crizotinib and does not target c-MET at relevant doses (75). ASCEND-4 was the first phase III trial to compare a next generation ALK TKI, ceritinib, against chemotherapy, ultimately demonstrating improved PFS (76). Patients with asymptomatic BM were included and intracranial responses were substantial, with icORR of 72.7% and median duration of response (DOR) of 16.6 months. In the ASCEND-1, ASCEND-2, and ASCEND-5 trials, ceritinib was evaluated in ALK+ NSCLC patients with prior platinum-based chemotherapy and/or crizotinib. Intracranial outcomes were less favorable with icORR ranging about 35%-55% (77–79). ASCEND-7 was a phase II trial specifically assessing outcomes of patients with BM treated with ceritinib. Patients with prior RT and ALKi, prior ALKi only, prior RT only, and RT/ALKi-naïve, respectively, had icORRs of 39.3%, 27.6%, 28.6%, and 51.5%, respectively (80).

Similarly, alectinib is a next generation ALK TKI active against crizotinib-resistant tumors that has improved CNS efficacy (81). In the phase III ALEX trial comparing alectinib versus crizotinib in treatment naïve ALK+ NSCLC, the 21 patients with BM had impressive icORR of 81% and DOR of 17.3 months. A *post-hoc* analysis of the J-ALEX study by Nishio et al., which included ALK+ NSCLC patients who were untreated or with at most one prior chemotherapy regimen, demonstrated that alectinib reduced the risk of CNS progression by approximately 50% and 20% in patients with and without baseline BM, respectively (82). The cumulative incidence rate of CNS progression in patients with baseline BM was low at 5.9% at one year with alectinib versus 16.8% with crizotinib. Even in the pretreated setting following platinum agents or crizotinib, subgroup analysis of the ALUR trial and several phase II trials showed icORR ranging 50-75%, icDCR as high as 80-100%, and DOR of approximately 11 months (59–62, 83).

More recently, brigatinib and ensartinib are second generation ALK TKIs that have shown superior efficacy over crizotinib in the phase III ALTA-1L and eXalt3 clinical trials, respectively (72, 73). In patients with ALK TKI-naïve NSCLC with BM, brigatinib had an intracranial ORR 78% with a long duration of response of 27.9 months (72). In a phase II dose-escalation trial in patients who progressed on crizotinib, the icORR was more modest with a higher dose demonstrating an icORR of 67% and median PFS of 12.8 (58) In the eXalt3 trial, ensartinib found an intracranial icORR of 64% and icDCR of 100% in ALK TKI-naïve patients, similar to results of early phase clinical trials with pretreated patients who had icORRs of 64-70% and icDCR of 93-98% (56, 57, 73).

Lorlatinib is a third generation ALK-TKI developed to overcome the main limitations of second generation ALK TKIs, namely, additional secondary ALK resistance mutations and limited CNS penetration (84). Updates from a phase I/II trial by Bauer et al. and Felip et al. evaluating intracranial outcomes following prior ALK TKI inhibitor demonstrated substantial but decreasing efficacy with subsequent lines of therapy (63, 64). Lorlatinib had icORR, icDCR, and median DOR of 87.5%, 100%, and NR respectively in patients treated with crizotinib only, 66.7%, 66.6%, and 20.7 months respectively in patients treated with one second generation ALK TKI, and 54.2%, 87.4%, and 23.4 months is patients treated with more than one 2^nd^ gen ALK TKI. A phase II study conducted by Dagogo-Jack et al. enrolled 23 patients on a second generation ALK TKI who had developed CNS only progression and discovered that lorlatinib provided an icORR of 59%, icDCR of 95%, and median PFS of 24.6 months (65). In the phase III CROWN trial evaluating loratinib in the upfront setting, loratinib produced excellent icORR of 82% in 17 patients with BM, 71% of whom had complete intracranial responses (71).

Development of newer generations of ALK TKIs have resulted in superior efficacy in the upfront and refractory settings and have improved CNS penetration. Given the long natural history and younger age at diagnosis of ALK+ tumors, and enhanced intracranial activity of newer ALK TKIs, the question of whether ALK+ NSCLC BM can be initially managed with ALK TKIs while deferring upfront RT is pertinent. Crizotinib is not an ideal agent for the deferral of RT. Notably, in the pooled analysis of PROFILE 1005 and 1007, patients treated with crizotinib and who had no prior RT had a poor icORR of 18%, icDCR of 56% and median time to intracranial progression of 7 months (68). In the ASCEND-4 trial, evaluating ceritinib in the upfront setting, 13 patients with BM without prior RT had much improved icORR of 69.2% and icDCR of 92.3% (76). Additionally, ASCEND-7 reported less favorable icORRs of 51.5% and 27.6% in patients who were both RT/ALK TKI-naïve and had prior ALK TKI only, respectively (80). Gadgeel et al. conducted a pooled analysis of two phase II trials utilizing alectinib in crizotinib-refractory patients, reporting intracranial ORR and icDCR of 58.5% and 82.9%, respectively, in patients without prior RT (85). Ensartinib in a phase II trial demonstrated excellent 88% ORR in patients with prior RT versus 66% icORR in patients without (57).

Petrelli et al. conducted a pooled analysis of 21 studies involving 1,016 patients receiving crizotinib and 2^nd^ generation ALK-TKIs and found intracranial ORR and icDCR in the first-line setting were 39.2% and 70.3%, and in the ALK-TKI pretreated setting 44.2% and 78.2%, respectively (86). The authors note that icORR was not influenced by prior treatment with RT, and that icORR was 49% among those who had never received RT. A multi-institutional retrospective study comparing TKIs with or without RT for BM demonstrated that for the 53 ALK+ NSCLC patients included, there was no difference in time to intracranial progression or time to treatment failure, questioning the added benefit of RT (49).

## Other targeted therapies

KRAS mutations are present in approximately 22% of lung cancers, and yet historically targeted agents have failed to show clinical efficacy despite significant effort and multiple avenues of inhibition (87). KRAS is a GTPase, activation of which leads to downstream signaling that promotes cell growth and proliferation (88). More recently, the KRAS G12C mutation, which is present in nearly 40% of KRAS-mutated NSCLC, has been targeted pharmacologically. Sotorasib is a small molecule irreversible inhibitor of KRAS G12C and was the first targeted agent against KRAS approved by the FDA (89). A *post hoc* analysis of phase 1/2 CodeBreaK 100 trial by Ramalingam et al. evaluated sotorasib in the setting of pretreated KRAS G12C mutant NSCLC with BM and reported an icORR of 25%, icDCR of 77.5%, and median DOR of 11.1 months (90). 65% of patients had received prior radiation and 20% had resection. A newer agent, adagrasib, is currently being evaluated in the accruing KRYSTAL-1 trial, and early data from 25 patients demonstrate an intracranial ORR, icDCR, and median PFS of 31.6%, 84.2%, and median PFS of 4.2 months (91). 

Rearrangements of the receptor tyrosine kinase ROS1 is a driver in a subset of NSCLC, occurring in 1-2% of cases (92). ROS1 rearrangement is associated with lung cancer patients of a younger age, non- to light- smoking history, and adenocarcinoma histology. Neurotropism is high with BM estimated to be present in approximately 36% of patients (93). Due the multi-target nature of ALK inhibitors, crizotinib and next generation ALK TKIs are being utilized in the treatment of ROS1+ NSCLC, with observations being made that the CNS is frequently a site of progression in as many as 50% of ROS1+ NSCLC (93). In a phase 1/2 trial of loratinib in ROS1+ NSCLC, TKI-naïve patients had an intracranial ORR of 64% while patients treated previously with crizotinib had an icORR of 50% (94).

c-MET is a proto-oncogene encoding a receptor tyrosine kinase. Gene amplification or aberrant splicing leads to proliferation and metastasis of cancer cells (95). The prevalence of c-MET oncogene activation is approximately 5% with gene amplification and altered gene splicing comprising 1.4% and 3.3%, respectively (96). Wolf et al. evaluated capmatinib, a selective c-MET inhibitor with CNS penetration, in a phase I/II trial in NSCLC with either a c-MET exon 14 skipping mutation or c-MET amplification. They reported an intracranial ORR and icDCR of 53.8% and 92.3%, respectively in 13 patients (97). Another selective c-MET inhibitor, tepotinib, was evaluated in a phase II trial in patients with c-MET exon 14 skipping mutation and was found to have an icORR of 55% and median DOR of 9.5 months in a cohort of 11 patients (98).

RET is another oncogene encoding a receptor tyrosine kinase, and rearrangements are found in only 1-2% of NSCLC (99). RET rearrangements are associated with a younger age, non-smoking history, poorly differentiated tumors, and nodal involvement (100). Multi-kinase inhibitors targeting RET were initially used with unimpressive results. In a multi-institution database of patients with RET-rearranged NSCLC BM treated with multi-kinase inhibitors (including but not limited to cabozantinib, vandetanib, sunitinib, ponatinib, vandetanib, and alectinib), Drilon et al. reported a short median PFS of 2.1 months in 37 patients and an icORR of 18% in a separate cohort of 11 patients (101). More promising are selective RET inhibitors, two of which have been FDA approved for treatment of RET+ NSCLC. Selpercatinib was assessed in a phase I/II trial of patients with RET fusion+ NSCLC, some of whom had received platinum-based chemotherapy, and reported a remarkable icORR of 91% and median DOR of 10.1 months in a small cohort of 11 patients (102). In a phase I/II trial, patients with RET fusion+ NSCLC received pralsetinib, and in the 9 patients with measurable BM, icORR was 56% and median DOR was not reached (103).

The NTRK gene family (NTRK1, NTRK2, and NTRK3) encode neurotrophic receptor tyrosine kinases. Genomic rearrangements resulting in NTRK fusion oncogenes result in constitutively active proteins (104). The prevalence of NTRK fusion genes in NSCLC is <1%. Data regarding intracranial activity of targeted therapy against NTRK is developing. A recently updated analysis of 3 phase I/II studies including patients with NTRK fusion positive solid tumors treated with the multikinase inhibitor entrectinib enrolled 31 patients with NSCLC and baseline CNS disease and demonstrated an icORR of 64.5% (105, 106). Larotrectinib is a highly selective NTRK inhibitor that has shown efficacy in NTRK fusion positive lung cancers with CNS metastases; however, data evaluating intracranial control is not available (107).

Overall, the data for these molecular targets are limited and emerging. **Table 3** summarizes clinical outcomes reported by early phase studies and retrospective reviews by targeted therapy agent. Similar to EGFR-mutated and ALK-rearranged NSCLC, these molecular targets show high propensity for involvement and progression in the CNS. At this point, local therapy including resection and radiation will be integral for CNS control until longer term data with larger patient numbers mature.

**Table 3:** CNS-related Clinical Outcomes of Other Targeted Therapies and Immunotherapy in NSCLC with Brain Metastases.

Study	Population	N	Intervention	icORR	icDCR	mPFS (mo)	mTTP (mo)	mDOR (mo)
CodeBreaK 100	KRAS G12C+ NSCLC with BM s/p RT or resection	40	sotorasib	25%	77.50%	NA	NA	11.1
KRYSTAL-1	KRAS G12C+ NSCLC with untreated BM	25	adagrasib	31.60%	84.20%	4.2	NA	NA
(94)	ROS1+ NSCLC	35	loratinib	TKI naïve: 64%, prior crizotinib: 50%	NA	NA	NA	NR
(97)	NSCLC with a MET exon 14 skipping mutation or MET amplification	13	capmatinib	53.80%	92.30%	NA	NA	NA
(98)	NSCLC with MET exon 14 skipping mutation	11	tepotinib	55%	NA	NA	NA	9.5
(103)	NSCLC with RET fusion+	9	pralsetinib	56%	NA	NA	NA	NR
(102)	NSCLC with RET fusion+, untreated or s/p platinum agent	11	selpercatinib	91%	NA	NA	NA	10.1
(106)	NTRK fusion+ NSCLC with BM	31	entrectinib	64.50%	NA	NA	NA	NA
(108)	PD-L1 positive NSCLC with BM	37	pembrolizumab	30%	40.50%	NA	NA	5.7
(109)	NSCLC with BM	255	PD-1 and PD-L1 inhibitors	20.60%	43.90%	1.7	NA	NA
(110)	Squamous NSCLC with treated BM s/p one prior systemic therapy	38	Nivolumab	18.40%	47.30%	5.5	NA	NA
(111)	Non-squamous NSCLC s/p 1 prior systemic treatment	409	Nivolumab	16.60%	40%	NA	NA	NA
(112)	NSCLC with BM	11	pembrolizumab with or without chemotherapy	34.60%	63.70%	NA	NA	NA
(113)	NSCLC with BM with PD-L1 TPS ≥ 50%	23	pembrolizumab	treated BM: 77%, nontreated BMs: 60%	treated BM: 85%, nontreated BMs: 60%	treated BM: 13.6, nontreated BMs: 18.6	NA	NA
(114)	Non-squamous NSCLC with untreated BM	40	atezolizumab with carboplatin and pemetrexed	42.50%	90%	7.1	NA	NA

NSCLCl, non-small cell lung cancer; BM, brain metastases; icORR, intracranial overall response rate; icDCR, intracranial disease control rate; mo, month; TKI, tyrosine kinase inhibitor; mTTP, median time to progression; mPFS, median progression free survival; mDOR, median duration of response; NR, not reached; RT, radiation therapy; NA, not applicable.

## Immunotherapy

Immune checkpoint inhibitors have revolutionized the treatment landscape for advanced NSCLC. By targeting PD-1 and CTLA-4 on exhausted CD8+ T cells and PD-L1 on tumor cells, these drugs restore immune system surveillance and enhance antitumor activity (115). As a result, there is emerging data on intracranial efficacy of these agents. Pooled analysis of large randomized clinical trials involving pembrolizumab monotherapy and pembrolizumab/platinum-based chemotherapy combinations in advanced NSCLC patients with BM have demonstrated improvements in OS and PFS in general (116, 117). However, granular data on intracranial responses are lacking in these trials.

In a phase II trial of 42 patients with NSCLC with BM treated with pembrolizumab, intracranial ORR and icDCR was 30% and 40.5%, respectively, with a median duration of response of 5.7 months for patients with PD-L1 expression >1% (108). However, among the 5 patients with undetectable PD-L1, there was no response. Similarly, in a large cohort of 255 patients treated with PD-1 and PD-L1 inhibitors, Hendriks et al. noted an icORR of 20.6% and PFS of 1.7 months (109). Notably, this patient population was diverse, with positive PD-L1 expression in only 61.5% of patients and with 27.4% of patients receiving steroids prior to treatment. Through an expanded access program in Italy, a retrospective analysis of patients receiving nivolumab who had received at least one prior systemic therapy showed icORR of 16-18% and icDCR 43-47% (110, 111). However, in 23 patients with NSCLC with BM receiving pembrolizumab and PD-L1 tumor proportion score ≥ 50%, Wakuda et al. reported improved results, with icORR of 77% and 60% and PFS of 13.6 and 18.6 months in pretreated and non-pretreated BM, respectively (113). Additionally, a phase II trial by Nadal et al. yielded an intracranial ORR of 42.5% and icDCR of 90% with atezolizumab combined with chemotherapy in treatment-naïve non-squamous NSCLC patients (114). These studies suggest that immune therapy may be an option for some patients with BM, particularly those whose tumors have high levels of PD-L1-expression.

## Radiation necrosis risk with combined modality treatment

Radiation necrosis is characterized as injury to normal brain tissue months to years after radiation therapy (118). Depending on the location and severity, radiation necrosis can be morbid, potentially requiring pharmacologic and invasive interventions. The rates of radiation necrosis can be as high as 34% (119). Whether targeted therapy and immunotherapy increases the risk of radiation necrosis is an area of active inquiry. Miller et al. retrospectively reviewed a large NSCLC dataset of 2276 lesions in 826 patients and reported radiation necrosis rates for EGFR+ and ALK+ lesions of 7.6% and 17.3%, respectively (120). When compared to wildtype EGFR or ALK tumors, ALK+ but not EGFR+ lesions were associated with higher 12-month cumulative incidences of radiation necrosis. Notably, receipt of EGFR or ALK inhibitors did not significantly impact rates of radiation necrosis, suggesting that they may be safely administered with stereotactic radiosurgery (SRS).

Evidence evaluating immunotherapy and radiation necrosis risk are mixed and is primarily derived from retrospective cohort data. In a series of 480 patients in which NSCLC comprised 61% of the total cohort, Martin et al. reported radiation necrosis rates of 20% in patients receiving immunotherapy versus 6.8% without (121). After adjusting for tumor histology, receipt of immunotherapy was associated with a 2.5 times increased risk of radiation necrosis following SRS, with melanoma associated with the greatest risk of radiation necrosis. On the other hand, in a separate study evaluating the risk of RT-related adverse events in 163 patients with advanced NSCLC patients receiving SRS, partial brain irradiation or WBRT, Hubbeling et al. reported no differences in all-grade toxicity or grade 3 or higher toxicity with receipt of immunotherapy (122). Similarly, Chen et al. found that in 260 patients with 634 brain metastases from NSCLC (60%), melanoma (27%), and renal cell carcinoma (13%), administration of SRS and immunotherapy was not associated with acute neurologic toxicity (123). For further reading, Loganadane et al. provide a comprehensive review of radiation necrosis in NSCLC patients with a focus on targeted therapy and immunotherapy (118).

## Conclusion

BM are a significant source of morbidity and mortality in patients with oncogene driven NSCLC. These cancers have demonstrated a higher predilection for CNS involvement, and due to improved survival overall, control of CNS disease is paramount. Traditionally, local therapy including resection, WBRT and SRS has been the cornerstone of intracranial control in light of the limited CNS penetration of historical systemic therapy. The review of CNS ORR across multiple targetable mutation positive NCSLC subtypes show that while early agents had modest intracranial activity, successive generations of TKIs have improved upon this. In EGFR-mutant tumors, osimertinib has shown remarkable activity and may be considered for patients with limited BM, reserving radiosurgery for progression. Omission of upfront radiation is being tested in the accruing OUTRUN trial. Similarly, tumors with high levels of PD-L1 expression may respond to immune therapy. In ALK-positive and other rare oncogene-positive cancers, data are more limited but encouraging. A treatment strategy of systemic therapy alone with close imaging and follow-up could be considered for patients with small and asymptomatic BM and for whom the benefits of upfront, aggressive local therapy may not be readily apparent.

In summary, the management of BM is rapidly evolving with improvements in systemic therapies that have brain penetrance. Research is needed on the integration of and interactions between these systemic treatments with local therapies. Future clinical trials should prioritize marker driven studies, patient-centered quality of life outcomes, neurocognitive outcomes of systemic therapy alone compared to radiotherapy, and investigation of systemic agents as a sole modality stratified by disease burden and symptom acuity. Additionally, new imaging tools should be developed to predict CNS response to various treatments and refine assessment of therapeutic response.

## Author contributions

CB and JY conceived of and provided critical edits for the manuscript. CB performed literature searches and drafted the manuscript. ML, CC, DP, AT-T, TC, EM, SC, JS provided edits for the manuscript. All authors contributed to the article and approved the submitted version.
